# Adaptive antitumor immune response stimulated by bio-nanoparticle based vaccine and checkpoint blockade

**DOI:** 10.1186/s13046-022-02307-3

**Published:** 2022-04-08

**Authors:** Xuewei Bai, Yanmei Zhou, Yuki Yokota, Yoshihiro Matsumoto, Bo Zhai, Nader Maarouf, Hikaru Hayashi, Rolf Carlson, Songhua Zhang, Aryanna Sousa, Bei Sun, Hossein Ghanbari, Xiaoqun Dong, Jack R. Wands

**Affiliations:** 1 Liver Research Center, Rhode Island Hospital, Department of Medicine, The Warren Alpert Medical School of Brown University, RI 02903 Providence, USA; 2 Department of Pancreatic and Biliary Surgery, The First Affiliated Hospital of Harbin Medical University, Harbin, 150081 Heilongjiang Province People’s Republic of China; 3 Department of Anesthesiology, Key Laboratory of Hepatosplenic Surgery, Ministry of Education, The First Affiliated Hospital of Harbin Medical University, Harbin, 150081 Heilongjiang Province People’s Republic of China; 4 Department of Surgical Oncology and Hepatobiliary Surgery, The Fourth Affiliated Hospital of Harbin Medical University, Harbin, 150001 Heilongjiang China; 5 Currently at Athanor Biosciences Inc., Halethorpe, MD 21227 USA

**Keywords:** Aspartate β-hydroxylase (ASPH), Immune checkpoint inhibitor, Lambda phage vaccine, Triple negative breast cancer, Hepatocellular carcinoma, Metastasis, Tertiary lymphoid structures

## Abstract

**Background:**

Interactions between tumor and microenvironment determine individual response to immunotherapy. Triple negative breast cancer (TNBC) and hepatocellular carcinoma (HCC) have exhibited suboptimal responses to immune checkpoint inhibitors (ICIs). Aspartate β-hydroxylase (ASPH), an oncofetal protein and tumor associated antigen (TAA), is a potential target for immunotherapy.

**Methods:**

Subcutaneous HCC and orthotopic TNBC murine models were established in immunocompetent BALB/c mice with injection of BNL-T3 and 4 T1 cells, respectively. Immunohistochemistry, immunofluorescence, H&E, flow cytometry, ELISA and in vitro cytotoxicity assays were performed.

**Results:**

The ASPH-MYC signaling cascade upregulates PD-L1 expression on breast and liver tumor cells. A bio-nanoparticle based λ phage vaccine targeting ASPH was administrated to mice harboring syngeneic HCC or TNBC tumors, either alone or in combination with PD-1 blockade. In control, autocrine chemokine ligand 13 (CXCL13)-C-X-C chemokine receptor type 5 (CXCR5) axis promoted tumor development and progression in HCC and TNBC. Interactions between PD-L1^+^ cancer cells and PD-1^+^ T cells resulted in T cell exhaustion and apoptosis, causing immune evasion of cancer cells. In contrast, combination therapy (Vaccine+PD-1 inhibitor) significantly suppressed primary hepatic or mammary tumor growth (with distant pulmonary metastases in TNBC). Adaptive immune responses were attributed to expansion of activated CD4^+^ T helper type 1 (Th1)/CD8^+^ cytotoxic T cells (CTLs) that displayed enhanced effector functions, and maturation of plasma cells that secreted high titers of ASPH-specific antibody. Combination therapy significantly reduced tumor infiltration of immunosuppressive CD4^+^/CD25^+^/FOXP3^+^ Tregs. When the PD-1/PD-L1 signal was inhibited, CXCL13 produced by ASPH^+^ cancer cells recruited CXCR5^+^/CD8^+^ T lymphocytes to tertiary lymphoid structures (TLSs), comprising effector and memory CTLs, T follicular helper cells, B cell germinal center, and follicular dendritic cells. TLSs facilitate activation and maturation of DCs and actively recruit immune subsets to tumor microenvironment. These CTLs secreted CXCL13 to recruit more CXCR5^+^ immune cells and to lyse CXCR5^+^ cancer cells. Upon combination treatment, formation of TLSs predicts sensitivity to ICI blockade. Combination therapy substantially prolonged overall survival of mice with HCC or TNBC.

**Conclusions:**

Synergistic antitumor efficacy attributable to a λ phage vaccine specifically targeting ASPH, an ideal TAA, combined with ICIs, inhibits tumor growth and progression of TNBC and HCC.

**Supplementary Information:**

The online version contains supplementary material available at 10.1186/s13046-022-02307-3.

## Background

Cancer is a leading cause of mortality globally, accounting for an estimated 19.3 million new cases and 10.0 million deaths in 2020 [1]. By 2040, the global burden of cancer is expected to reach 28.4 million new cases with an associated 16.4 million deaths [1]. Despite advancements in surgery, chemotherapy, radiotherapy and targeted therapy, 830,180 people died of liver cancer (hepatocellular carcinoma [HCC], accounting for 85-90% of all cases), whereas 684,996 patients died of breast cancer in 2020 world-wide [1]. Early-intermediate stages HCC can be treated with locoregional ablation, resection or liver transplantation, whereas advanced stage HCC (~ 50%) lacks benefit from systemic therapy [2]. HCC is multi-drug resistant and notoriously aggressive, so improvement in clinical outcome is modest/incremental [2]. Triple negative breast cancer (TNBC) with absent expression of estrogen receptor, progesterone receptor and HER2/neu, accounting for 15-20% of all breast cancer cases and characterized by biological heterogeneity, has the least favorable prognosis [3]. TNBC represents a major unmet need attributed to a highly metastatic phenotype, with chemotherapy being the only available option. Therefore, it is essential to develop immunotherapy in an attempt to treat such aggressive and refractory cancers (in particular TNBC and HCC) with a dismal prognosis.

Immunotherapy has become the backbone of evolutionary cancer treatment. The burgeoning immune checkpoint inhibitors (ICIs), such as CTLA4, PD-1 and PD-L1 antibodies, in combination with immune stimulation supports that host immune system could serve as a therapeutic modulator for tumor development and progression. ICI blockade may modulate or reprogram cellular interactions in the complex tumor-microenvironment (TME) [4]. Mechanistically, the PD-1/PD-L1 pathway mediates tumor-induced immunosuppression [5]. Currently, PD-1/PD-L1 inhibitors are pharmacologically designed to prevent PD-1/PD-L1 interactions, thus releasing an adaptive immune response to attack tumor cells [6]. However, only a few types of cancers are susceptible to this approach. Thus far, ICIs have exhibited little or no activity in subsets of tumors with lower mutational burdens [7, 8]. Pembrolizumab was applied for treatment of advanced HCC. Unfortunately, a phase III clinical trial failed in HCC patients who had previously received Sorafenib [9]. Furthermore, most TNBC patients treated with ICIs in monotherapy exhibited primary immune resistance and early progression within the first 2–3 months, regardless of enrichment for PD-L1 positivity [3]. The discovery of target antigens may boost immune response if combined in a vaccine construct with checkpoint inhibitor blockade to achieve anti-tumor effects not present in resistant tumors. To fill in the gap in knowledge, we explore if ICIs integrated with a unique molecular targeted vaccine can be therapeutic for multi-drug resistant HCC (for identification-of-concept) and TNBC (for proof-of-concept).

Immune system is key in cancer control as demonstrated by the requirement for a pre-existing intratumor adaptive immune response for effective immunotherapies, such as ICIs. A new concept called immune contexture has been introduced to describe how different interconnected parts can be assembled and arranged in tumor milieu. Immune contexture [10–13] of complicated TME predicts clinical outcome of several types of cancers, which evaluates various components infiltrating the tumor as an integrated system and defines interconnected elements. Immune contexture is deciphered by immunoscores [14] of tumor infiltrating lymphocytes (TILS) based on (1) type (2); density (3); anatomic site (4); intratumoral location (5); composition of different subpopulations, especially CD8^+^/CD62L^+^ effector T and CD45RO^+^ (in human) or CD44^+^ (in mouse) memory T (6); functional orientation of specific secretome derived from Th1 cells (e.g., IFN-γ) and CTLs (e.g., granzymes, perforin, granulysin) (7); organizational pattern (scattered vs. clustered) and (8) spatial distribution (relationship with the tumor and adjacent vasculature/lymphatic system) of immune cells.

Another novel concept, tertiary lymphoid structure (TLS) has been proposed to explore correlation between adaptive immune response to comprehensive therapy and clinical outcome of cancer patients [15]. Generally, TLSs are ectopic aggregates indicating cellular neogenesis in non-lymphoid tissues at the site of inflammation and tumor growth [15–17]. Tumor associated TLSs are typically located peritumorally with morphological, cellular and molecular similarity to secondary lymphoid organs, such as lymph nodes [15–17]. Generally, TLSs are composed of a T cell zone containing mature DC-Lamp+ dendritic cells (DCs), adjacent to a B cell zone which includes a typical germinal center (GC) comprising B cells embedded within a network of follicular DC (FDC) [13]. PNAd+ high endothelial venules (HEV) surrounding TLS [18] enable lymphocyte entry to and exit from blood vasculature. We hypothesize that the presence and nature of TLSs predict tumor responses to immunotherapy.

Targeted therapy, as a cornerstone of precision medicine, has become the focus of anticancer drug development. We have identified ASPH, a transmembrane β-hydroxylase [19], highly expressed during embryonic development but silenced at birth and during adulthood. It is only reactivated to imitate oncogenesis [19, 20], translocated to cell membrane, where N and C-terminal regions are exposed to the extracellular environment. Subsequently, ASPH interacts with immune system and serves as a tumor associated antigen (TAA) stably presented on a broad spectrum of tumor tissue types and represents an ideal target for immunotherapy [21]. Importantly, antigenic epitopes that reside on the extracellular regions of ASPH efficiently stimulate antigen specific T-cell responses to a variety of expressing tumor tissue types [22]. Previous studies have established that ASPH is a target for immunotherapy using a dendritic cell (DC) vaccine approach in syngeneic animal models of HCC and cholangiocarcinoma [23, 24]. ASPH promotes tumor cell proliferation, migration, invasion [20, 25] and metastasis [26–28]. Its oncogenic properties [28–31] are attributed to: 1) activation of Notch [32, 33] and SRC [28] cascades; 2) anti-apoptosis; 3) dysregulation of cell cycle control; 4) dysregulation of senescence; and 5) gain-of cancer stemness. These processes are necessary and sufficient for spontaneous development and progression of HCC in a double transgenic murine model [34]. Therefore, targeting this oncogenic network has therapeutic potential [35]. Immunotherapy is particularly attractive approach since ASPH [20–24, 28–33]: 1) is highly expressed on cell surface in various malignancies; 2) expresses at extremely low or negligible levels in normal human tissues except in placenta (a highly invasive tissue); 3) has a defined pro-oncogenic role; 4) approximately 85% of hepatitis C virus and hepatitis B virus related HCC, as well as 90% of breast cancers, and especially in TNBC and HER2 amplified subtypes [32], exhibits ASPH upregulation [36]. In addition, moderate to high level expression of ASPH portends a worse surgical outcome [36], and predicts a more aggressive clinical course [36, 37]. ASPH overexpression correlates with early disease reoccurrence, and subsequently reduced overall/disease-free survival [32].

Phage display is a nanotechnology that allows the isolation of high-affinity ligands for diverse substrates. This system can provide personalized cancer therapy depending on unique and functional targets [38], such as ASPH. Bacteriophages, belonging to viruses that infect bacterial hosts, are candidates for vaccine delivery because of high stability in harsh environments and ease for large-scale production. Importantly, bacteriophages can serve as potent adjuvants to enhance the host responses to immunizing antigens [39, 40]. Bacteriophages favorably display and present peptides to the immune system. Notably, lambda (λ) phage contains a linear double-stranded DNA (dsDNA) genome, which can be modified for antigen display, an advantage over filamentous phages [41]. Because λ phage is assembled in the cytoplasm, it is particularly well-suited for the display of cytoplasmic proteins and has the capability for multi-antigen presentation which may allow for further antigen selection and amplification [42]. A newly developed ASPH-based λ phage vaccine has promising immunostimulatory effects [21]. This novel construct expresses multiple copies of ASPH peptides on the surface of immunogenic λ phage particles, thereby eliciting an effective immune response. The ASPH-based λ phage display has great potential for developing new strategies of vaccine discovery and production [43]. This is a novel approach for a vaccine platform targeting a broad spectrum of ASPH expressing tumors. Here, to provide unique opportunities for tailoring precision therapy, we employ an ASPH-based λ phage vaccine construct when used in combination with checkpoint inhibitors produces a more robust synergistic anti-tumor immune response in pre-clinical murine models of HCC (subcutaneous, rapid growth, extremely difficult to treat) for identification-of-concept and TNBC (orthotopic, developing spontaneous metastases) for proof-of-concept, two types of cancers with a long-standing paucity of effective therapies and particularly poor outcomes.

## Methods

### Cell lines

The murine breast tumor cell line 4T1 (ATCC, CRL-2539) and liver HCC cell line BNL 1ME A.7R.1 (BNL) (ATCC, TIB-75) were cultured at 37 °C in a humidified atmosphere containing 5% CO_2_ in Dulbecco’s modified Eagle’s medium (DMEM) supplemented with 2 mM L-glutamine, 10% FBS and antibiotics (penicillin and streptomycin).

### Recombinant human ASPH protein preparation

The full length human *ASPH* gene (GenBank accession No. S83325) was cloned into the EcoRI site of the pcDNA vector (Invitrogen, Carlsbad, CA). Recombinant ASPH protein (rASPH) was produced in a Baculovirus Expression system (Invitrogen) according to manufacturer’s instructions [22–24, 43].

### Construction of bacteriophage λ to display ASPH peptides [43]

The ASPH-based λ phage vaccine (bio-nanoparticle based therapeutic vaccine [BNP-TV]) was developed and synthesized in collaboration with investigators at Panacea Therapeutics [43]. Bacteriophage λ was designed to display ASPH protein fused at the carboxyl terminus of its capsid protein gpD [43]. This system applied both to the N and C-terminal regions of human ASPH protein, designated as ASPH construct λ1 and λ3, respectively. The N- and C-segments were amplified from *hASPH* gene using specific oligo primers by PCR, with sequences modified to produce restriction sites for Nhe I and Bssh II enzymes. After restriction enzyme digestion, the *ASPH* segments were inserted at the NheI-BsshII site of the 3′ end of DNA segment encoding gpD controlled by the Lac Operon. These constructs were established in donor plasmid pVCDcDL1A harboring *loxPwt* and *loxP511* sequences. Subsequently, Cre-expressing *E. coli* was transformed with recombinant plasmids and infected with recipient λ phage DL1 (λ-DL1) carrying DNA segment flanked by *loxPwt* and *loxP511* sites. Recombination in vivo at the *loxP* sites and cointegration with *Ampr* spontaneously lysed the *E. coli,* which released assembled “virions” to culture media. This cointegration produced recombinant λ phage to display ASPH peptides fused at the C-terminus of gpD. Recombination with unmodified pVCDcDL3 yielded control phage particles displaying no peptide [43].

### Reagents

In vivo monoclonal antibody (mAb) anti-mouse PD-1 (CD279) (clone RMP1-14) was purchased from Bioxcell (Catalog #BE0146, West Lebanon, NH). The antibody was administered intraperitoneally (i.p.) either alone or in combination with λ phage vaccine.

### Murine subcutaneous model of HCC and orthotopic model of TNBC

**Fig. 1:**
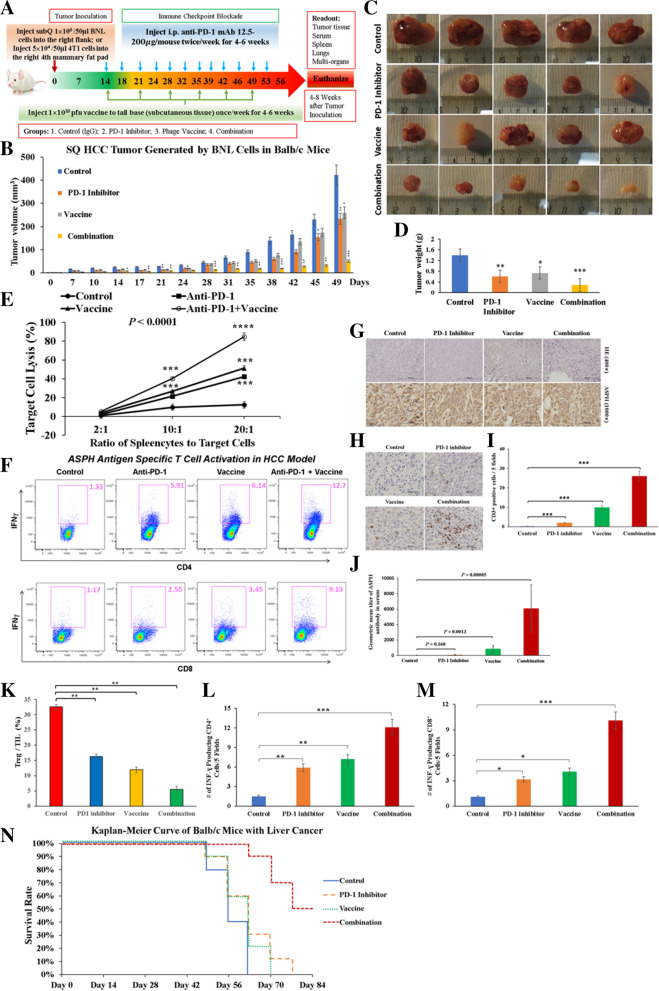
Therapeutic effects of different treatments on tumor development and progression in a murine subcutaneous model of HCC. **A** Experimental protocols for murine subcutaneous model of liver (HCC) and orthotopic model of breast cancer (TNBC) generated by injecting ASPH-expressing BNL cells subcutaneously and 4T1 cells orthotopically (mammary fat pad), respectively. For each model, Balb/C mice were randomly divided to 4 groups (*n* = 6/group): control, PD1 blockade (anti-PD-1 mAb), ASPH-based λ phage vaccine, and combination (PD-1 blockade + vaccine). **B** Growth curves of HCC tumors. Note the substantial anti-tumor effect of combination therapy (also see Fig. S1B). **C** Macroscopic appearance of resected HCC tumors. **D** Tumor weights of HCC after 49 days in different groups. **E** In vitro cytotoxicity of splenocytes derived from mice against target BNL cells. **F** Antigen (ASPH) specific CD4^+^ and CD8^+^ T cell activation as demonstrated by upregulation of IFN-γ. **G** Histological features of ASPH expression in HCC tumors. **H-I** Number of CD3^+^ tumor infiltrating lymphocytes (TILs) in HCC tumors. **J** Anti-ASPH antibody titers in serum derived from different groups. **K** Percentage of immunosuppressive CD4^+^/CD25^+^/FOXP3^+^ Tregs among TILs in response to therapy. **L-M** Number of IFN-γ producing CD4^+^ and CD8^+^ T cells, respectively, in response to therapy. **N** Overall survival of mice harboring HCC in response to therapy. ^*^, *p* < 0.05; ^**^, *p* < 0.01; ^***^, *p* < 0.005; ^****^, *p* < 0.001

This study had been approved by the Institutional Animal Care and Use Committee at Rhode Island Hospital (Providence, RI). The experimental design is shown in Fig. 1A. Female immunocompetent BALB/c mice aged 6-8-weeks-old (Charles River Laboratories, Wilmington, MA) were randomly assigned to 4 groups for each model (*n* = 10/group): control (empty phage construct + IgG isotype antibody), PD-1 blockade (PD-1 inhibitor), vaccine (immunized with ASPH-based λ phage), and combination (PD-1 blockade + vaccine). BNL (1 × 10^6^) or 4T1 (5 × 10^4^) cells in 50 μl (volume of cell suspension: Matrigel at 1:1 ratio) were subcutaneously inoculated into the right flank or the 4th mammary fat pad, respectively. Therapeutic schedule starting in 2 weeks after BNL or 4T1 cells were implanted, when the tumor was established. The mice in the vaccine and combination groups were immunized once a week by subcutaneous injection 1 × 10^10^ pfu λ1 phage particles (expressing N-terminal human ASPH peptides) suspended in 50 μl of sterile saline into the base of tail. Simultaneously, in PD-1 blockade and combination groups, anti-PD-1 mAb (12.5 μg-200 μg/mouse) was i.p. administered twice per week. Tumor size was measured [24, 43] using calipers 2–3 times per week and tumor volume was calculated as (length×width^2^)/2).

Six to eight weeks (for HCC model) or 4 weeks (for TNBC model) after tumor cell inoculation, mice bearing tumors with the shorter diameter exceeded 10 mm were euthanized according to specific requirements from Animal Welfare Committee of the Rhode Island Hospital (Providence, RI). Primary tumors, spleen, lungs and other involved tissue samples were harvested, fixed or stored at − 80 °C. The number and size of macro-metastatic lesions in the lungs (immersed in Bouin’s fixative solution) were counted and measured, respectively. Tumors were excised and fixed with 10% formalin for hematoxylin & eosin (H&E) and immunohistochemistry (IHC) staining.

### Isolation and characterization of murine splenocytes [43]

Following euthanasia, spleens were resected from mice bearing tumors in different groups and dispersed into cell suspension using the plunger end from 3 ml syringe, followed by a filtering process with 70 μm cell strainer. Erythrocytes were lysed with RBC lysis solution buffer (BD Biosciences, San Jose, CA). After isolation, splenocytes (1 × 10^6^ cells/ml) were cultured in RPMI-1640 (containing 10% FBS, 1× non-essential amino acids, 1× amino acid solution, 100 μM sodium pyruvate, 1× penicillin-streptomycin and 2-Mercaptoethanol [50 μM]) and re-stimulated with 0.4-0.5 μg/ml of recombinant protein (rASPH) and 2 × 10^8^ pfu/ml λ phage particles in vitro for 4 days [24, 43]. Simultaneously, 10 ng/ml of IL-2 (PeproTech, Rocky Hill, NJ) was added for 2 days. Following the 4-day re-stimulation of splenocytes with λ phage and rASPH, the supernatants derived from culture media were collected for Enzyme-Linked Immunosorbent Assay (ELISA) to quantify cytokine secretion. This assay was previously optimized for T cell activation using DCs pulsed with ASPH containing microparticles [22, 24]. To remove dead cells from re-stimulated splenocytes, density-gradient centrifugation was performed using Lympholyte®-M (Cedarlane, Burlington, NC). Purified re-stimulated splenocytes were used for in vitro cytotoxicity and flow cytometric analyses.

### In vitro cytotoxicity

Briefly, BNL or 4 T1 cells were seeded into a 96-well plate and allowed to attach for 1 h. Subsequently, suspension of splenocytes derived from mice in different groups was added at a ratio of splenocytes to target (BNL or 4 T1) cells varying from 2:1, 10:1 to 20:1, respectively, and incubated with BNL cells for 4 h. Lactate dehydrogenase (LDH) release from the lysed BNL cells was measured as an indicator of cytotoxic activity with Cytotoxicity Detection Kit^PLUS^ (LDH) (Sigma-Aldrich, #4744926001) [24, 43]. All experiments were performed in triplicate.

### Flow cytometry

After isolation and re-stimulation, the splenocytes were collected, washed and immunophenotyped by using the following anti-mouse mAbs: CD3-eFluor 450, CD4-FITC (Thermo Fisher Scientific), and CD8a-APC-H7 (BD Biosciences). Appropriate isotype controls were used for comparison. After staining, the splenocytes were assessed by BD FACSAria™ II Flow Cytometer (BD Biosciences, San Jose, CA, USA). Dead cells were excluded by using LIVE/DEAD Fixable Dead Cell Stain (Invitrogen). Antigen specific CD4^+^ or CD8^+^ T cells were analyzed with FlowJo software (Tree Star Inc., Ashland, OR) [43]. All experiments were performed in triplicate.

### Elisa

Whole blood (400 μL) was collected from the mouse’s heart and centrifuged at 3000 rpm for 20 min. Serum samples were collected and stored at − 80 °C. Interferon gamma (IFN-γ) ELISA Kit (Thermo Fisher Scientific, #KMC4021) was used to measure secretion levels in cell culture supernatants after serial dilutions (1:4). A total of 100 μl of standard, control or serum/supernatant was added to each well of a 96-well plate and incubated for 2 h at room temperature. The solution was thoroughly mixed and washed 4 times with 1× PBS buffer/well. Then, 100 μl mouse IFN-γ biotin conjugate solution was added into each well and incubated for 1 h at room temperature. Subsequently, 100 μl 1× streptavidin-HRP solution was added into each well and incubated for 30 min at room temperature. Afterwards, each well was washed and added with 100 μl stabilized chromogen. The absorbance was read at 450 nm after adding stop solution. The concentration of each sample was calculated according to the standard curve. All experiments were performed in triplicate.

### IHC & immunofluorescence (IF)

Tumor cells were seeded on coverslips embedded with glycine, fixed with 4% formaldehyde and stained. Tumor tissues were fixed in 4% formaldehyde, embedded in paraffin, and sectioned into 4-μm-thick slides. The antigen was retrieved, and endogenous peroxidase activity was quenched through a 30-min treatment in methanol with 3% hydrogen peroxide. Non-specific antigen was blocked for 1 h. The slides were incubated with primary antibodies overnight at 4 °C. After rinsing, HRP (Vector Laboratories, #PK4001 and #PK-6102) or fluorescent second antibody (Thermo Fisher Scientific, #RMG101 and #A24538) was added onto the slides and incubated for 1 h. For IF, the slides were mounted with DAPI (Vector Laboratories, #H-1800) and observed under the fluorescence microscope. For IHC, the slides were incubated with peroxidase-label for 30 min, developed with DAB Kit (SK-4100, Vector Laboratories) and counterstained with hematoxylin. For quantitation, 5 microscopic fields were randomly selected at 400× magnification. Images were analyzed by NIH ImageJ software (https://imagej.nih.gov/ij/).

### Characterization of tertiary lymphoid structures (TLSs)

The existence of intra-tumoral TLSs was assessed morphologically on H&E stained FFPE slides [44]. IHC-stained cells were counted semi-quantitatively (scored at 0, 1, 2, 3, and 4 for none, very low, weak, intermediate, and high density of positive cells, respectively) in each intermediate-power field (IPF) in intra- and peri-tumoral areas of the entire section (original magnification × 100), and expressed as mean scores ± SEMs per IPF. The numbers of DC-LAMP^+^ mature DCs and CD8^+^ T cells were counted and expressed as mean ± SEMs per IPF. Immunostaining of CD4, CD8 and memory T cell marker (CD44 in mouse whereas CD45RO in human) was semi-quantified as a percentage of positive cells among CD3^+^ T cells. Necrosis was scored as the percent of the positive necrotic areas among the entire tumor section. Immunostaining and scoring were evaluated independently by 3 observers.

### Evaluation of tumor microenvironment

After deparaffinization, antigen retrieval and endogenous peroxidase blocking, immunohistochemical staining was performed using the VECTASTAIN Elite ABC kit (Vector Laboratories, Burlingame, CA) according to the manufacture’s protocol. Primary antibodies were against ASPH (laboratory developed mouse monoclonal antibody [mAb], diluted at 1:10000), PD-1 (ab214421), PD-L1 (ab213480), CD21 (ab75985) and CD19 (ab203615) purchased from Abcam; CD3 (MA5-14524), CD8 (14-0808-82), CD44 (A700-038), BCL-6 (PA5-14259), DC-LAMP/CD208 (PA5-96217), LRP1 (37-7600) and TLR4 (710185) from purchased from Thermo Fisher Scientific; CD25 (39475S) and FOXP3 (12653S) from Cell Signaling Technology. The FFPE sections were incubated with primary antibodies at 4 °C overnight, then color development was performed using DAB Peroxidase Substrate (Vector Laboratories) for 60–90 s. Counterstaining was performed by hematoxylin. The CD3^+^ and CD8^+^ T cells were counted per randomly selected 5–10 high-power fields (400×) and CD44^+^ cells were counted in 10 fields (1000×) using NIH ImageJ software (Bethesda, MD). The mean number of CD3^+^, CD8^+^ or CD44^+^ T cells infiltrating the tumor parenchyma from 5 different sections derived from 3 to 5 tumors were calculated.

### Statistical analysis

Statistical analysis was performed using SPSS software (version 22.0). Quantitative data was presented as means ± SEM and analyzed by one-way analysis of variance (ANOVA) (comparison among multiple groups, with Tukey post hoc or Kruskal–Wallis test, or with Dunn post hoc test for nonparametric comparison) as well as Student’s *t,* unpaired Mann–Whitney *U* or v Fisher’s Exact tests when appropriate (comparison between two groups). Equality of variance was examined using F-test. For tertiary lymphoid structures (TLS) assessment, immunostaining was manually annotated. IHC scores from different groups were compared by χ^2^ or Fisher’s Exact tests. A *p*-value of < 0.05 (two-sided) was considered as statistically significant.

## Results

### ASPH-MYC axis upregulates PD-L1 surface expression on HCC cells

In previous studies, we have demonstrated that ASPH activates the Notch cascade and upregulates downstream target genes, in particular c-MYC, to participate in oncogenic process [30]. Notably, c-MYC upregulates PD-L1 expression to promote tumorigenic immune evasion [45, 46]. Consequently, mice harboring untreated HCC or TNBC demonstrate upregulation of the PD-L1 by the ASPH-MYC signaling cascade (Supplemental Fig. S1A).

### Phage vaccination targeting ASPH in combination with PD-1 blockade strikingly reduces HCC tumor growth and progression in a syngeneic subcutaneous murine model

Combination therapy resulted in a substantial reduction in HCC development and growth compared to control. Tumor growth was blunted by either vaccine or PD-1 blockade alone, to an intermediate extent between control and combination therapy (Supplemental Fig. S1B; Fig. 1B-D). A substantial decrease in tumor volume of the excised HCC was attributed to combination therapy. Very little, if any, growth of the HCC was observed in response to combination therapy. In these tumors, 90% of the resultant tissue contained broad areas of necrosis and inflammation and very little viable tumor (data not shown).

### Antigen specific activation of CD4^+^ Th1 and CD8^+^ CTLs was stimulated by ASPH-based λ phage vaccine immunization in combination with PD-1 blockade in the BNL derived HCC model

To achieve anti-tumor responses, both CD4^+^ Th1 and CD8^+^ CTLs are involved [23, 24]. Thus, in vitro cytotoxicity assay was performed to measure CD8^+^ CTL function [24, 43]. There was a marked increase in CTL activity of splenocytes derived from the combination group when compared to control. Either anti-PD-1 or vaccine administration alone generated an intermediate response. The ASPH-based phage vaccination was more effective than PD-1 blockade with respect to lysis of BNL cells (Fig. 1E).

To validate the specificity of CTLs against ASPH as a target, in vitro cytotoxicity of splenocytes derived from the HCC model was evaluated in the context of 4 T1 (which induced metastatic breast cancer) as target cells with endogenous expression of ASPH. Splenocytes derived from combined treatment of HCC substantially enhanced CD8^+^ CTL activity against 4 T1 cells as compared to splenocytes derived from control animals (Supplemental Fig. S1C). Thus, splenocytes sensitized to ASPH in vivo in HCC can lyse target cells derived from other tumors (such as TNBC) that endogenously express ASPH.

To evaluate the adaptive immune response, the proportion of ASPH specific CD4^+^ Th1 or CD8^+^ CTL activated in splenocyte population was analyzed by flow cytometry (Fig. 1F). The number of ASPH specific CD4^+^ Th1 or CD8^+^ CTL was substantially increased as demonstrated by enhanced secretion of IFN-γ after restimulation of splenocytes with ASPH-based λ phage vaccine and rhASPH protein. The highest level of response was observed with combination therapy, compared to either λ phage vaccine or PD-1 blockade alone.

To explore dynamic changes in phenotypes, H&E and IHC were performed. Histologic/pathologic features of HCC varied significantly when comparing control to the other groups. However, ASPH was expressed robustly and at equal levels in tumors derived from all groups (Fig. 1G). More important, infiltration of CD3^+^ T lymphocytes into HCC tumor area was gradually increased from PD1 blockade, to vaccine, to combination therapy, as compared to control (Fig. 1H). Thus, combined therapy had exerted more profound anti-tumor effects than vaccine or PD-1 blockade alone (Fig. 1I). Importantly, anti-ASPH specific antibody (as an indicator of B cell response) could also be detected in vaccine and combination groups as expected, compared to control and PD-1 treated animals (Fig. 1J). Combination therapy significantly reduced infiltration of immunosuppressive CD4^+^/CD25^+^/FOXP3^+^ Tregs (Fig. 1K), whereas increased IFN-γ producing CD4^+^ and CD8^+^ cells, compared to other groups (Fig. 1L-M). Consequently, combination therapy substantially prolonged overall survival of mice with HCC (Fig. 1N).

### ASPH-MYC signaling upregulates PD-L1 expression on 4T1 breast cancer cells

Consistent with previous studies [45, 46], in mice harboring TNBC generated by 4T1 cells, PD-L1 was upregulated in association with ASPH-MYC [30] expression as demonstrated by IHC (Supplemental Fig. S2A). In this context, extensive metastatic spread to multi-organ was observed (Supplemental Fig. S2B).

### Phage vaccination targeting ASPH combined with PD-1 blockade significantly reduces TNBC tumor metastasis

**Fig. 2:**
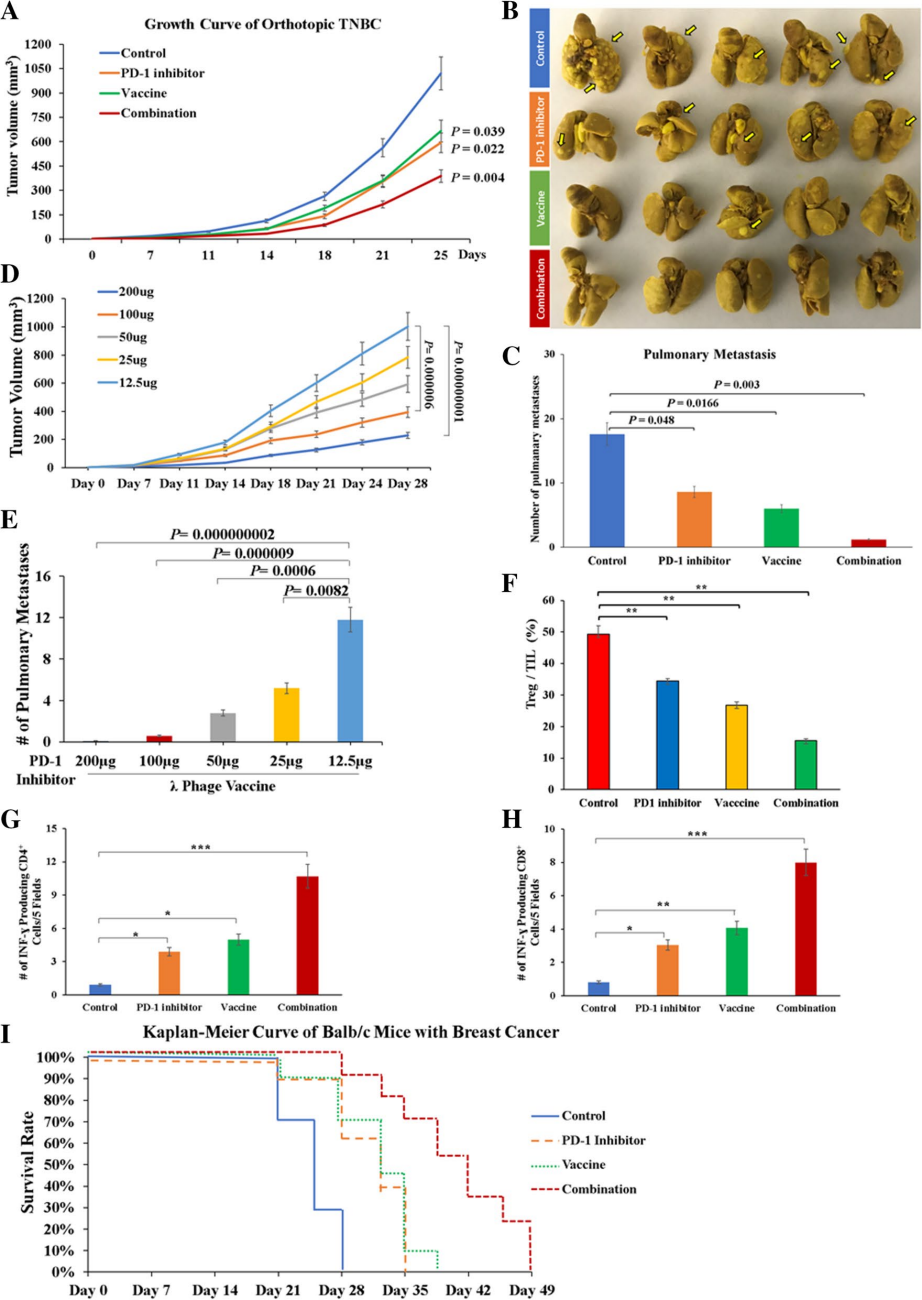
Anti-tumor effects of different reagents on tumor development and metastasis in an orthotopic model generated by 4T1 cells in Balb/C mice. **A** Growth curves of primary TNBC tumors. **B** Gross appearance of lungs harboring metastatic TNBC tumors. **C** Number of pulmonary metastatic tumors in mice derived from different groups. **D** Dose-dependent inhibitory effects of anti-PD-1 mAb on TNBC tumor growth in λ phage vaccinated mice as compared to the low dose group (12.5 μg), ^***^ *p* < 0.001; ^****^p < 0.000000001. **E** Dose-dependent inhibitory effects of anti-PD-1 mAb on pulmonary metastases in λ phage vaccinated mice. Comparisons were made to 4 different concentrations of anit-PD-1 mAb. **F** Percentage of immunosuppressive CD4^+^/CD25^+^/FOXP3^+^ Tregs among TILs in response to therapy. **G-H** Number of IFN-γ producing CD4^+^ and CD8^+^ T cells, respectively, in response to therapy. **I** Overall survival of mice harboring TNBC in response to therapy. ^*^, *p* < 0.05; ^**^, *p* < 0.01; ^***^, *p* < 0.005; ^****^, *p* < 0.001

The TNBC model had demonstrated widespread metastasis to different and distant sites, such as liver, lymph nodes, spleen, diaphragm, adrenal gland, and kidney (Supplemental Fig. S2B-D). Primary tumor growth was inhibited to a marked extent (65-70%) by treatment in the combination group (Fig. 2A). Combination therapy significantly reduced total metastatic tumor burden produced compared to control (Supplemental Fig. S2E-F). Notably, combination therapy inhibited multiple-organ metastases of TNBC, in particular pulmonary lesions (Fig. 2B-C). This anti-tumor effect was dose-dependent on the concentration of anti-PD-1 mAb administered (Fig. 2D). The high dose (200 μg) anti-PD-1 mAb demonstrates the most pronounced inhibitory effects on both size of primary tumor growth and number of pulmonary metastasis (Fig. 2E), compared to a low dose (12.5 μg). Combination therapy significantly reduced infiltration of immunosuppressive CD4^+^/CD25^+^/FOXP3^+^ Tregs (Fig. 2F), whereas increased IFN-γ producing CD4^+^ and CD8^+^ cells, compared to other groups, compared to other groups (Fig. 2G-H). Consequently, combination therapy substantially prolonged overall survival of mice with HCC (Fig. 2I).

### Antigen specific activation of CD8^+^ CTL and CD4^+^ Th1 following λ phage immunization in combination with PD-1 blockade

**Fig. 3:**
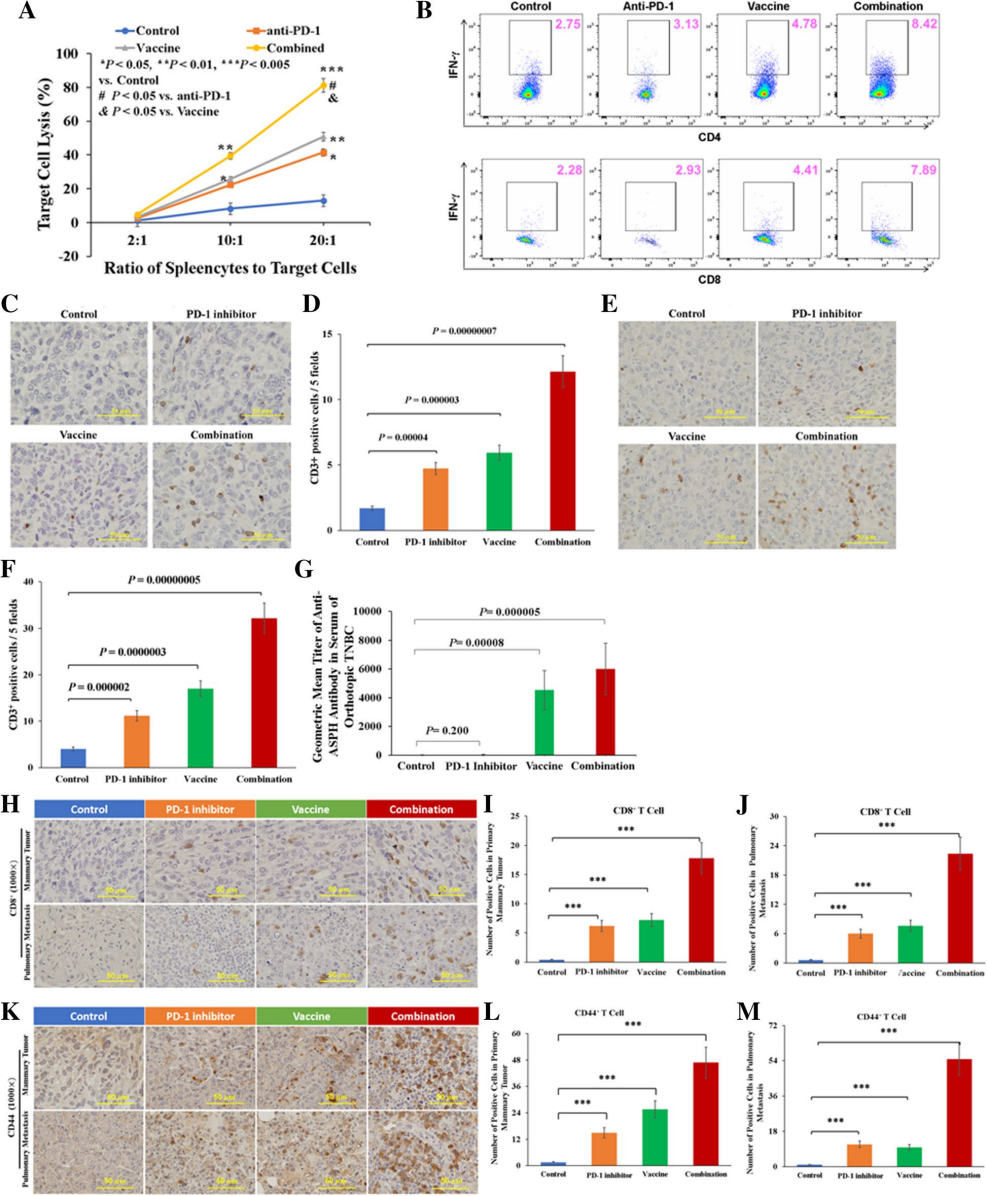
Activation of antigen specific TILs and B cells in a spontaneously metastatic model of TNBC. **A** In vitro cytotoxicity of splenocytes towards 4T1 cells. **B** Generation of antigen (ASPH) specific CD4^+^ and CD8^+^ T cells as demonstrated by upregulation of IFN-γ. **C-D** Number of CD3^+^ TILs in TNBC primary tumors and **E-F** pulmonary metastases. **G** ASPH specific antibody titers in serum derived from different groups. **H-J** Number of CD62L^+^/CD8^+^ effector CTLs in primary tumors and pulmonary metastases. **K-M** Number of CD44^+^ memory CTLs in primary tumors and pulmonary metastases. ^*^, *p* < 0.05; ^**^, *p* < 0.01; ^***^, *p* < 0.005; ^****^, *p* < 0.001

There was an increase in ASPH-specific CTL activity of splenocytes when compared to unvaccinated control. Administration of either anti-PD-1 mAb or vaccine alone generated intermediate responses. ASPH-based λ phage vaccination was as effective as PD-1 blockade with respect to CTL activity against 4 T1 cells (Fig. 3A). The percentage of antigen specific CD4^+^ or CD8^+^ T cells activated in a splenocyte population was analyzed by flow cytometry (Fig. 3B). Either vaccine alone or combination therapy led to a substantial increase in ASPH specific CD4^+^ and CD8^+^ activity as measured by the secretion of IFN-γ after prior restimulation of splenocytes with phage vaccine and rhASPH protein. The highest level of activity occurred in the combination group compared to either vaccine or PD-1 blockade alone. Combination therapy induced a substantial increase in infiltration of CD3^+^ T cells into the primary breast tumors that was produced by either PD-1 blockade or vaccine alone as compared to control (Fig. 3C-D). Likewise, combination therapy reduced pulmonary metastasis, compared to either vaccine or PD-1 blockade alone (Fig. 3E-F). Importantly, anti-ASPH specific antibody, as an index of B cell response, was detected in vaccine and combination groups (Fig. 3G). Combination therapy contributed to enhanced infiltration of CD8^+^ effector CTLs (Fig. 3H-J) and CD44^+^ memory CTLs (Fig. 3K-M) among the CD3^+^ TILs into both primary tumors and pulmonary metastases.

### Characterization of tumor microenvironment

In response to combination therapy, CD3^+^ TILs, including CD62L^+^ or CD8^+^ effector CTLs and CD44^+^ memory CTLs, were predominantly localized in the intra- and peritumoral TLSs [15] in close proximity to CD4^+^BCL-6^+^ T follicular helper cells (TFH) (Supplemental Fig. S3A-C), CD19^+^ B cell infiltrates (germinal center) (Fig. S3D-F), CD21^+^ follicular dendritic cell (FDC) [47] (Fig. S3G-J). This TLS could facilitate activation and maturation of DCs highly expressing LRP1^+^, TLR4^+^ or LMP3 (Supplemental Fig. S4A-J) in response to combination therapy. In most tumors treated with combination therapy, TILs surrounded the central B cell area and at the tumor-normal tissue interface, suggesting that TLSs are actively recruiting immune subsets to TME [15].

### CXCL13-CXCR5 interactions with PD1/PD-L1 inhibitory signal in HCC and TNBC

**Fig. 4:**
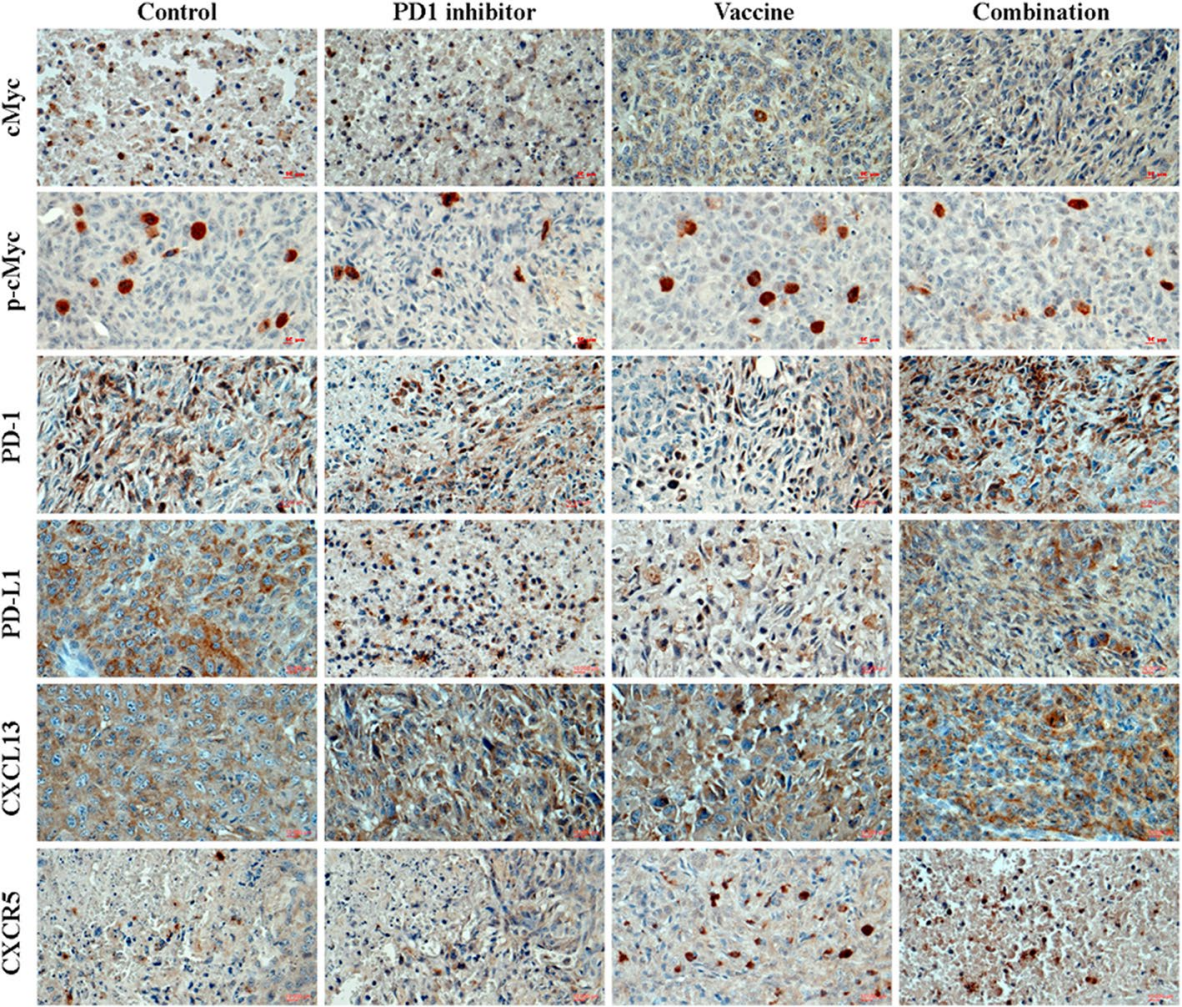
Expression profiling of major components involved in PD-1/PD-L1 inhibitory signal and CXCL13-CXCR5 axis in HCC. Expression profiling of MYC, activated (phosphorylated) MYC, PD-1, PD-L1, CXCL13 and CXCR5 in HCC tumor cells or stroma cells

**Fig. 5:**
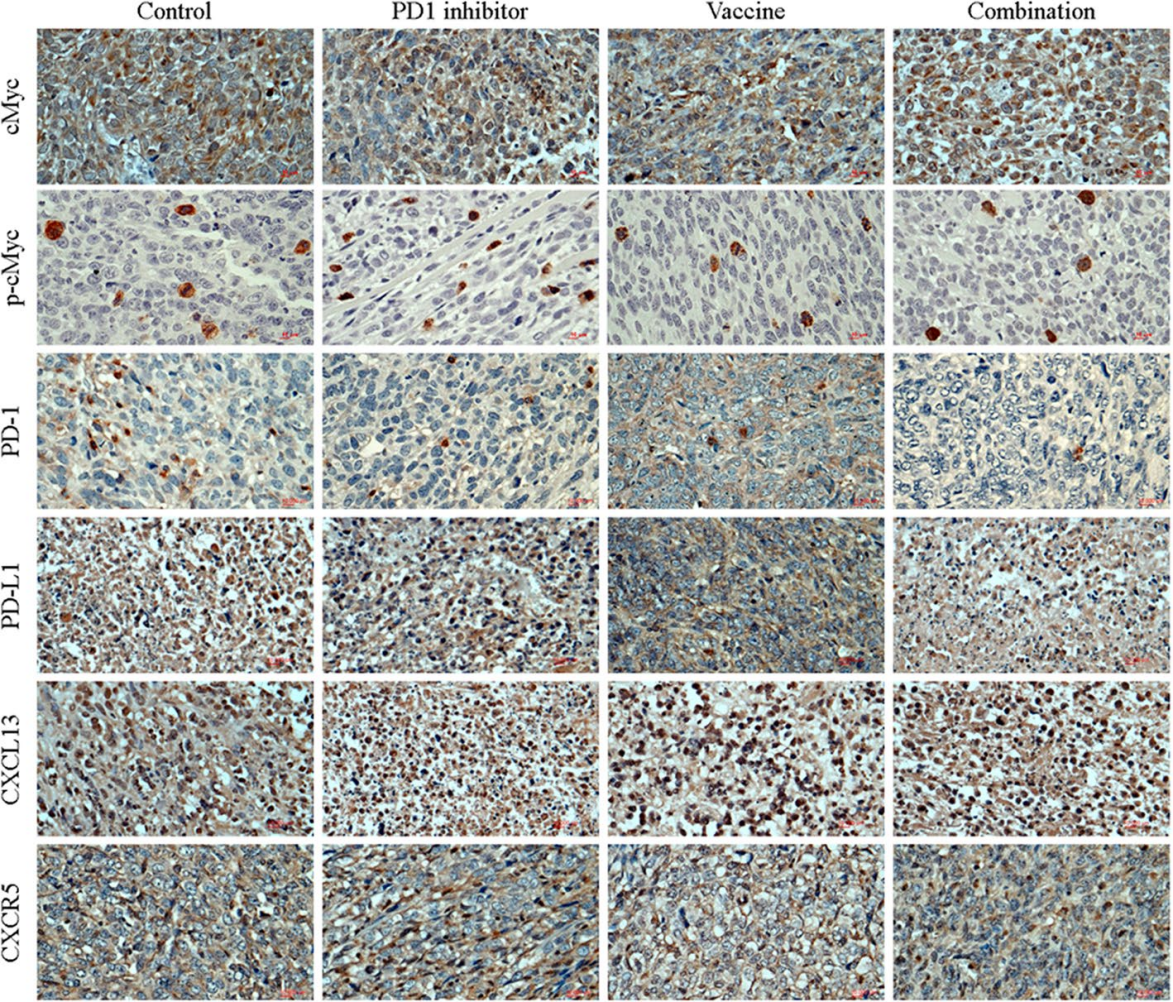
Expression profiling of major components involved in PD-1/PD-L1 inhibitory signal and CXCL13-CXCR5 axis in TNBC. Expression profiling of MYC, activated (phosphorylated) MYC, PD-1, PD-L1, CXCL13 and CXCR5 in TNBC tumor cells or stroma cells

**Fig. 6:**
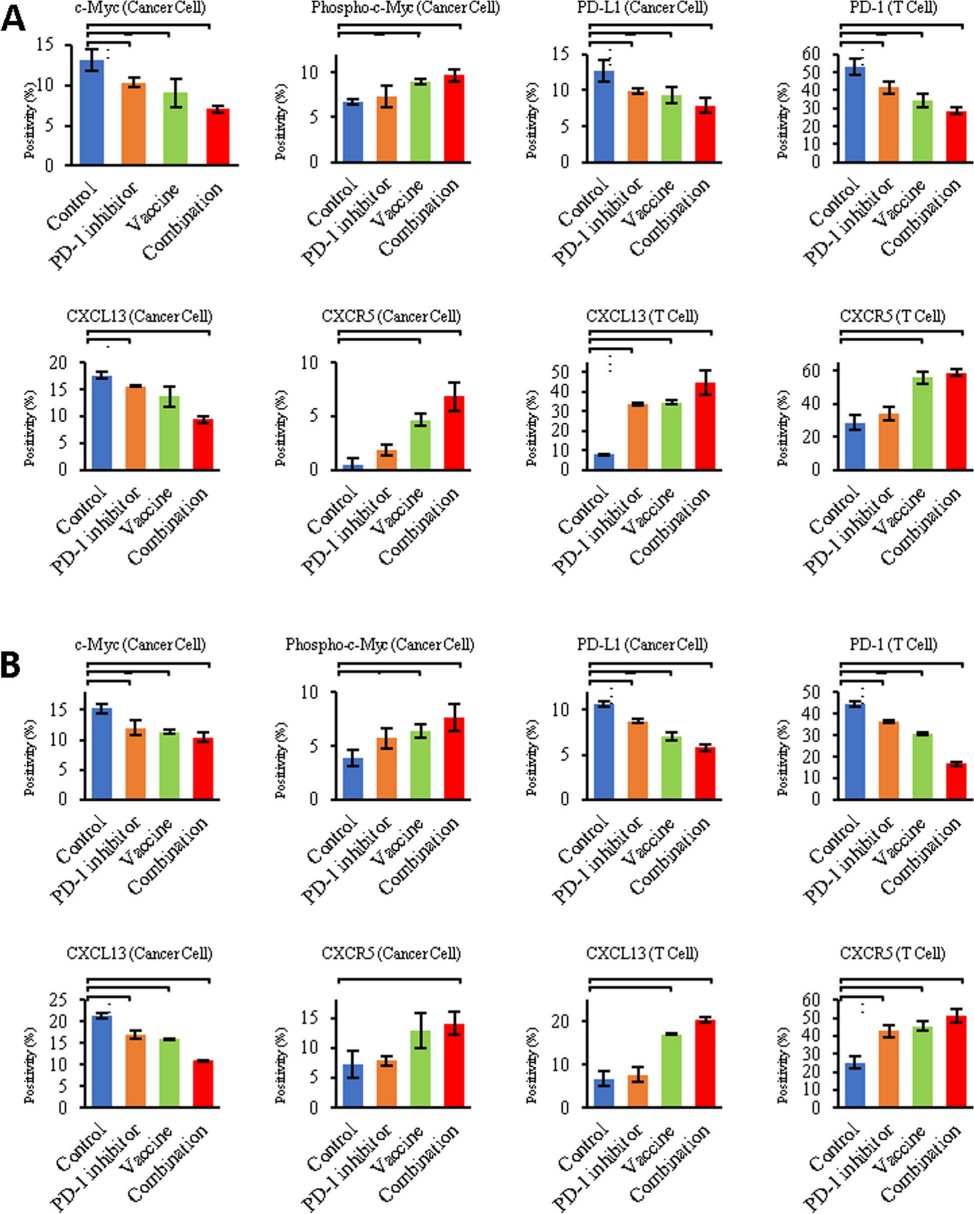
Expression profiling of major components involved in PD-1/PD-L1 inhibitory signal and CXCL13-CXCR5 axis in HCC and TNBC. As stratified by therapeutic strategies, differential expression levels of MYC, activated (phosphorylated) MYC, PD-1, PD-L1, CXCL13 and CXCR5 can be detected in (**A**) HCC and (**B**) TNBC tumor cells or stroma cells (TME). ^*^, *p* < 0.05; ^**^, *p* < 0.01; ^***^, *p* < 0.005; ^****^, *p* < 0.001

In HCC (Fig. 4**;** Fig. 6A) and TNBC (Fig. 5**;** Fig. 6B), PD-L1 was upregulated on cancer cells whereas PD-1 was upregulated on TILs [48]. In the control group, ASPH induced MYC activation to mediate PD-L1 upregulation. It was hypothesized that PD-L1^+^ cancer cells interacted with PD-1^+^ TILs, leading to exhaustion and apoptosis of TILs in TME.

Chemokine C-X-C motif ligand 13 (CXCL13) and its receptor, the G-protein coupled receptor (GPCR) CXCR5, play fundamental roles in inflammatory, infectious and immune responses [49]. We hypothesized that CXCL13-CXCR5 axis orchestrates tumor cell-stroma cell interactions that regulate functions of TILs within the TME, thereby modifying tumor development and progression, as well as determining responsiveness to immune-targeted therapies. As indicated by in vivo models, CXCL13-CXCR5 axis promotes cancer growth and metastatic dissemination (Figs.4 and 5). In TNBC and HCC, cancer cells would exhibit more aggressive phenotypes partially attributed to CXCL13-CXCR5 interactions in autocrine (to enhance self-renewal and survival through CXCL13 production and CXCR5 upregulation), paracrine (to induce proliferation and invasion signal for adjacent cancer cells) or endocrine (to facilitate pre-metastatic niche formation at distant sites) manners. The PD-1^+^ TILs that secreted CXCL13 and avidly interacted with CXCR5^+^PD-L1^+^ cancer cells were predominant in controls. However, PD-1^+^ TILs had impaired effector functions compared to PD1^−^ TILs (Supplemental Fig. S5A; Fig. 6). Thus, complex network involving tumoral and stromal (immune) CXCL13 and CXCR5 integrate to promote cancer cell autonomous and non-autonomous responses, highlighting autocrine, paracrine and endocrine interactions in dictating aggressive cancer phenotypes.

However, application of immunotherapy revealed upgraded complexity of CXCL13-CXCR5 axis. Upon combination treatment, PD-L1/PD-1 inhibitory signal and exhaustion of CTLs were substantially attenuated. In response to combination therapy, CXCL13 produced by ASPH^+^ expressing HCC or TNBC cell was capable of recruiting immune cells, especially CXCR5^+^/CD8^+^ TILs (including a proportion of CTLs), to participate in forming TLSs and to execute cytotoxicity against cancer cells. Accordingly, CXCL13 produced by CXCR5^+^/CD8^+^ TILs (through autocrine, paracrine or endocrine) might directly bind and induce cytotoxicity to lyse CXCR5^+^ tumor cells. Simultaneously, CXCL13 produced by CD8^+^ TILs could chemoattract and recruit CXCR5^+^ B and T_FH_ cells to jointly construct TLSs. This unique immune contexture characterized by TLSs confers sensitivity to combination therapy. Notably, upon combination therapy, ASPH-MYC and downstream PD-L1/PD-1 signals were blocked; CTLs secreted CXCL13 could bind to CXCR5 on cancer cells and produce cytotoxicity against them efficiently (Supplemental Fig. S5B; Fig. 6). These findings might decipher TLS, a unique immune cell architecture of the TME in HCC and TNBC with implications for previously uncharacterized roles of CXCL13-CXCR5 axis, which could act as a double-bladed sword in tumor development/progression and response to immunotherapy.

## Discussion

We have demonstrated that a λ phage vaccine construct expressing a N-terminal peptide of ASPH when combined with a checkpoint inhibitor substantially reduces both primary tumor growth and multi-organ metastasis in murine models of TNBC and HCC. The gpD fusion proteins of λ phage head are connected by linker peptide to N-terminal region of ASPH, which allows this λ phage to display TAA that can be presented or cross-presented to the host immune system [50]. This platform enables multiple copies of antigenic epitopes to be stably and efficiently displayed on λ phage head, even taken into account with low-affinity protein-protein interactions [50]. We have recently identified anti-tumor effects of this λ phage vaccine that targets ASPH expressing HCC [43]. In this setting, recombinant λ phage primes strong CD8^+^ CTLs responses both in vitro and in vivo against ASPH epitopes. This vaccine also activates Th1 response and elicits efficient production of antigen-specific antibodies without the addition of any adjuvant. Taken together, this ASPH-based λ phage vaccine in combination with PD-1 blockade produces a synergistic, potent anti-tumor immune response in syngeneic murine models of TNBC and HCC.

**Fig. 7:**
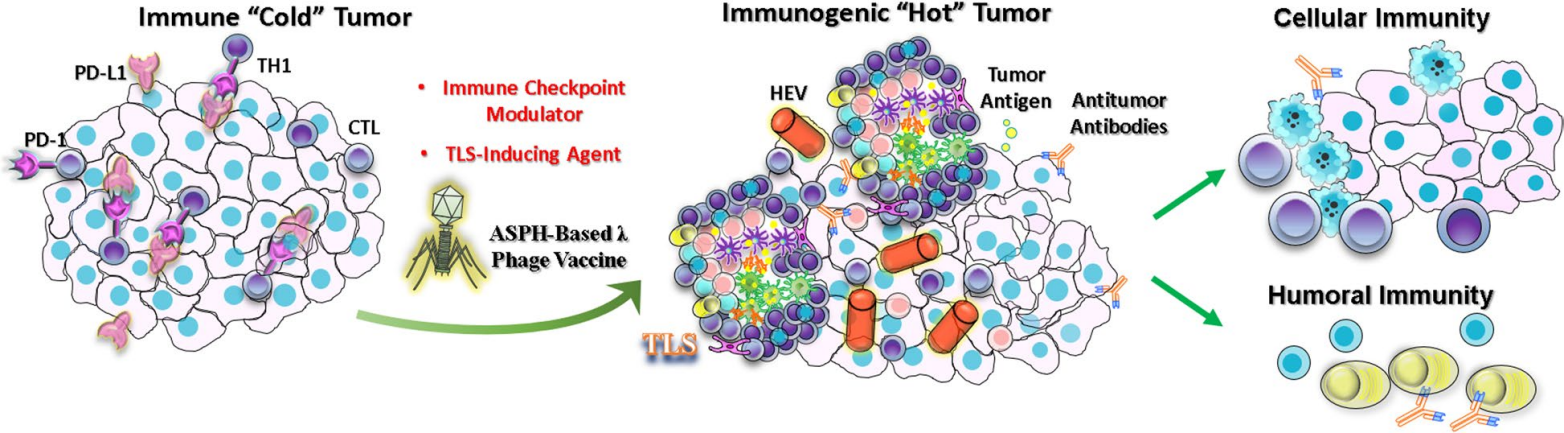
Hypothesized role of ASPH as a target for immunotherapy in HCC and TNBC. Immunotherapy manipulates composition and function of TLSs to reverse detrimental fate. ASPH-MYC axis upregulates PD-L1 on cancer cells and DCs. PD-1 is upregulated on exhausted TILs in immunosuppressive TME. Thus, immune cold/desert HCC and TNBC can be converted to immunogenic hot tumor (a phenotype characterized by TLS) in response to ASPH targeted immunotherapy combined with PD-L1/PD1 inhibitors, where ASPH may replace PD1/PD-L1 to serve as a surrogate biomarker for immune checkpoint inhibitor indication

Based on our findings, TLSs are indicated as a major player in antitumor immune responses. The presence and composition of TLS in TNBC and HCC models have been explored. How TLS interacts with cellular immune components of the host to initiate anti-tumor response has been explored. ASPH, an ideal TAA, serves as a target for immunotherapy in HCC and TNBC, which is partially dependent on TLSs [15] (Fig. 7). Ectopic TLSs, similar in architecture to secondary lymphoid organs, are classically defined as lymphoid aggregates forming in non-hematopoietic organs in response to chronic and non-resolving inflammatory processes, such as infection, graft rejection, autoimmune disease and more recently, cancer. Spontaneous TLSs have been documented in melanoma, lung, colorectal and breast cancer. TLSs are generally indicative of a reduced risk of recurrence and a favorable clinical outcome [11, 44, 51]. TLSs are proposed to provide a local and essential microenvironment for generating anti-tumor cellular and humoral immune responses, linked with improved prognosis [11, 44]. Interestingly, TLSs are principally involved in antigen-specific anti-tumor immune responses by promoting induction of effector and central memory T cells and plasma cells. However, the role of TLSs in HCC pathogenesis is controversial as they might promote the growth of hepatocyte progenitor cells [52]. Intra-tumoral TLSs correlated with an increased risk of late tumor relapse and portended a trend toward shortened survival after surgical resection [52]. In a murine model of chronic NF-κB activation, TLSs were composed of a micro-niche to facilitate the growth of malignant hepatocyte progenitor cells through activation and secretion of pro-tumoral cytokines [52]. Therefore, the pathological significance of TLSs in TNBC and HCC needs to be further explored in pre-clinical animal models and patients.

In this study, intra-tumoral TLSs correlated with a better therapeutic response to vaccine immunization in combination with PD-1 blockade. Thus, TLSs could represent the existence of an in situ, active and effective anti-tumor immunity [53]. Moreover, high endothelial venules (HEVs) in malignant tissue promotes anti-tumor immunity by recruiting naive lymphocytes to TLS in TME, followed by extranodal priming of effector cells. However, TLSs and chronic intratumoral inflammation might also correlate with a tolerogenic TME and tumor aggressiveness [54, 55].

Immune check inhibitors such as nivolumab, an anti-PD-1 antibody, have become a choice for HCC patients who have been previously treated with multi-kinase inhibitor sorafenib. Intra-tumoral infiltration by immune cells may serve as a predictor of sensitivity to PD-L1/PD-1 checkpoint inhibitors, as these drugs may enhance in situ anti-tumor responses and overcome immune evasion. Consistently, intra-tumoral TLSs are sensitive to immunomodulating therapeutic strategies [53]. Importantly, ASPH activates Notch signaling to upregulate MYC expression, which in turn upregulates PD-L1 [45, 56]. The MYC-mediated activation of PD-L1 gene could enable HCC and TNBC cells to escape from immune surveillance [56]. In addition, PD-L1 expression was upregulated on tumor cells by signaling through ASPH-Notch-MYC in both murine TNBC and HCC animal models. More importantly, PD-1 blockade was effective when administered in combination with a λ phage vaccine that targeted epitopes residing in the N-terminal peptide of ASPH. This finding suggests that immunosuppressive TME can be reprogrammed to introduce T cell penetration and to execute cytotoxicity against cancer cells [57–59].

Chemokines regulate the infiltration immune cell subsets into tumors, which can edit tumor immunity and influence therapeutic outcomes in patients. In addition, chemokines target interacted tumor-TME, to regulate cancer cell proliferation, survival, stemness and angiogenesis [60]. CXCL13 was identified as a B-cell chemoattractant. CXCL13/CXCR5 axis exerts key functions in lymphoid neogenesis, and has been implicated in the pathogenesis of various autoimmune, inflammatory and lymphoproliferative diseases. Production of CXCL13 within the tumor milieu is proposed to impact proliferation, migration, and invasive properties of cancer cells [61]. In our study, CXCL13/CXCR5 axis interacts with PD-1/PD-L1 inhibitory signal and acts a double-edged sword in tumor progression and response to immunotherapy. In the absence of PD-1 inhibitor, CXCL13 ligand secreted by cancer cells binds to CXCR5 receptors and promotes an aggressive tumor phenotype. Simultaneously, CXCL13 derived from cancer cells binding CXCR5 on T cells may enhance PD1/PD-L1 mediated exhaustion resulting in apoptosis of effector lymphocytes (Fig. S5). In the presence of PD-1 inhibitor, PD-L1 (on cancer cells)/PD-1 (on T cells) inhibitory signal is blocked. Effector T cells are activated and survive dependent on the autocrine CXCL13/CXCR5 axis. T cells secrete CXCL13 to recruit CXCR5^+^ immune cells to the TME, thus initiating jointly anti-tumor immune responses. In response to combinatory therapy, cancer cells are subject to apoptosis when exposed to CXCL13^+^ T cells [49, 60]. Dissecting sophisticated molecular and signaling networks regulated by CXCL13-CXCR5 axis and how this chemokine-receptor controls dynamic interactions between TNBC/HCC cell and the TME will be critical to identify novel effectors and therapeutic targets for cancer treatment.

Bacteriophages are critical constituents of normal human body with a vital role in bacterial population dynamics and host immune response modulation. Bacteriophage-based anticancer therapy has become robust with advanced phage display technology [38]. This λ phage vaccine could be employed for both cancer prevention and treatment. It is designed to specifically target ASPH using bacteriophage surface-expressed ASPH N-terminal peptides bound by a linker. This formulation is highly immunogenic to overcome self-antigen tolerance by providing a novel antigen presentation mode with inherent adjuvant properties. Vaccination with a λ phage-displaying ASPH peptides has various advantages. First, multiple copies of peptides are displayed on a single λ phage head particle. Once the initial phage display has been made and stocks have been stored, subsequent production is straightforward and less complicated than having to produce coupling peptides to carriers. Phage-displayed peptides can get access to both MHC class I and II pathways. Thus, λ phage display vaccines can stimulate both cellular and humoral immune responses. Although as extracellular antigens, the majority of immune responses will be antibody biased. Particulate antigens, including phages, can access the MHC class I pathway through cross-presentation and priming. This process is likely to be involved for stimulating an adaptive cellular immune response by CD8^+^ T cells to eliminate tumor cells. This λ phage can also act as a nonspecific immune stimulator attributed to a combination of foreign DNA (possibly due to CpG motifs) and repeating peptide motif coating the phage head.

In preclinical murine models, both BNL and 4 T1 cell lines produce robust growth when implanted subcutaneously or orthotopically into syngeneic BALB/c mice. Inoculated animals rapidly develop HCC and TNBC and have to be euthanized as early as 5–6 weeks or 3-4 weeks, respectively, after implantation due to a large tumor burden with poorly differentiated histology [24]. Endogenous expression of ASPH in BNL or 4 T1 induced tumor remains in the same range as in naturally developed human HCC or TNBC [24]. Using HCC and TNBC murine models, a λ phage vaccine construct which displays the N-terminal ASPH peptide modestly inhibits subcutaneous tumor growth and progression. However, antitumor effects are substantially amplified by a simultaneously administered checkpoint PD-1 inhibitor. A prophylactic immunization schedule could be envisioned prior to surgical resection of HCC or TNBC tumor mass to prevent early disease reoccurrence and to inhibit the development and progression of established micro-metastatic disease. We hypothesize that residual tumor cells that may exist following surgery could be abolished or reduced by the host immune responses generated from this λ phage vaccine and checkpoint inhibitor given in combination.

## Conclusions

Taken together, this combination immunotherapy stimulates an adaptive immune response to a single chemically defined antigen that is highly expressed on cellular surface of a wide majority of malignancies. This combination strikingly reduces metastases to various regions, particularly the lungs which was unanticipated. Such combination therapy may have wide application to various solid tumors, such as liver, pancreas, gastric, esophageal, breast, cholangiocarcinoma and sarcomas since these tumors highly express ASPH.

Collectively, a λ phage vaccine targeting ASPH combined with a checkpoint inhibitor successfully initiates antigen specific CD8^+^T and B cell-mediated cellular and humoral immunity in HCC and TNBC. This approach overcomes immunologic tolerance since ASPH is an oncofetal protein and thus an ideal TAA. We are led to believe that combined therapy allows ASPH peptides to be recognized, taken up, processed and presented as well as cross-presented by DCs since this λ phage acts as an adjuvant to display tumor restricted molecules such as ASPH. These findings have potential for a potent and broad-spectrum approach to a wide variety of aggressive solid human tumors.

## Supplementary Information

**Additional file 1: Figure S1.** Effect of ASPH upregulation on PD-L1 expression. (A) PD-L1 upregulation was associated with ASPH-MYC signaling in BNL HCC derived tumors. (B) HCC tumor size at 49 days after implantation of BNL cells in different groups. ASPH-MYC signaling was observed in tumors derived from different groups. (C) In vitro cytotoxicity of splenocytes derived from mice of HCC model against 4T1 breast cancer target cells.

**Additional file 2: Figure S2.** The TNBC exhibits an aggressive metastatic phenotype. (A) PD-L1 expression was associated with activation of ASPH-MYC signaling in TNBC cells. (B) Evidence of multi-organ metastases (lymph nodes, liver, spleen, pancreas, diaphragm, adrenal gland and kidney) in mice bearing TNBC tumors. (C) Representative histologic features of metastatic tumors. (D) Total number, (E) Frequency and (F) Distribution (number of mice with vs. without metastasis) of metastatic lesions or animals who developed metastases from different groups in TNBC model. ^*^, *p* < 0.05; ^**^, *p* < 0.01; ^***^, *p* < 0.005; ^****^, *p* < 0.001.

**Additional file 3: Figure S3.** Intra-tumor TLS in 4T1 TNBC breast cancer tumors. (A-C) Number of CD4^+^/BCL6^+^ T follicular helper cell (TFH) population located in primary tumors and mediastinal metastases. (D-F) Number of CD19^+^ B population in primary tumors and pulmonary metastases. (G-J) Number of CD21^+^ follicular dendritic cell (FDC) population in primary mammary tumors as well as mediastinal and pulmonary metastases. ^*^, *p* < 0.05; ^**^, *p* < 0.01; ^***^, *p* < 0.005; ^****^, *p* < 0.001.

**Additional file 4: Figure S4.** Activation and maturation of DCs are attributed to TLSs. (A-C) Number of LRP1^+^ DCs population in primary tumors and mediastinal metastases. (D-F) Number of TLR4^+^ DCs population in primary tumors and pulmonary metastases. (G) Intra-tumoral TLSs in primary mammary tumors and metastases induced by combination therapy. (H) Peri-tumoral TLSs (colocalized with CD3^+^ TILs) induced by combinatory therapy. (I) LAMP3^+^/LRP1^+^/TLR4^+^ DCs population in primary tumors and pulmonary metastases. (J) LRP1^+^/ TLR4^+^ DCs population in hilar/mediastinal lymph nodes metastases. ^*^, *p* < 0.05; ^**^, *p* < 0.01; ^***^, *p* < 0.005; ^****^, *p* < 0.001.

**Additional file 5: Figure S5.** Hypothesized role of CXCL13/CXCR5 axis. (A) In the absence of PD-1 inhibitor, (autocrine, paracrine or endocrine) CXCL13 ligand secreted by cancer cells binding CXCR5 receptor to enhance aggressive phenotypes. Simultaneously, CXCL13 derived from cancer cells binding CXCR5 on T cells to aggravate PD1/PDL1 mediated T cell exhaustion, resulting in apoptosis of effector T lymphocytes. (B) In the presence of PD1 inhibitor, PD-L1 (on cancer cells)/PD-1 (on T cells) inhibitory signal is blocked. Effector T cells are activated and survive partially dependent on autocrine CXCL13/CXCR5. T cells secrete CXCL13 (paracrine or endocrine) to recruit CXCR5^+^ immune cells, thus initiating anti-tumor immune response. As attacked by CXCL13^+^ (paracrine) T cells, CXCR5^+^ cancer cells are subject to apoptosis.

## Data Availability

The datasets used and/or analyzed during the current study are available from the corresponding author on reasonable request.

## Abbreviations

ASPH: aspartate β-hydroxylase
CTL: cytotoxic T lymphocyte
CXCL13: Chemokine ligand 13
CXCR5: C-X-C chemokine receptor type 5
DC: dendritic cell
FDC: follicular dendritic cell
GC: germinal center
HEV: high endothelial venules
HCC: hepatocellular carcinoma
IHC: immunohistochemistry
IPF: intermediate-power field
PD-1: programmed cell death protein 1
PD-L1: programmed death-ligand 1
TAA: tumor associated antigen
TFH: follicular B helper T cell
TIL: tumor infiltrating lymphocyte
TLS: tertiary lymphoid structures
TME: tumor-microenvironment
TNBC: triple-negative breast cancer
