# Supplementary figures and images for: Adaptive antitumor immune response stimulated by bio-nanoparticle based vaccine and checkpoint blockade
Source: J Exp Clin Cancer Res. 2022 Apr 8;41:132. doi: 10.1186/s13046-022-02307-3 (PMC8991500; doi:10.1186/s13046-022-02307-3)

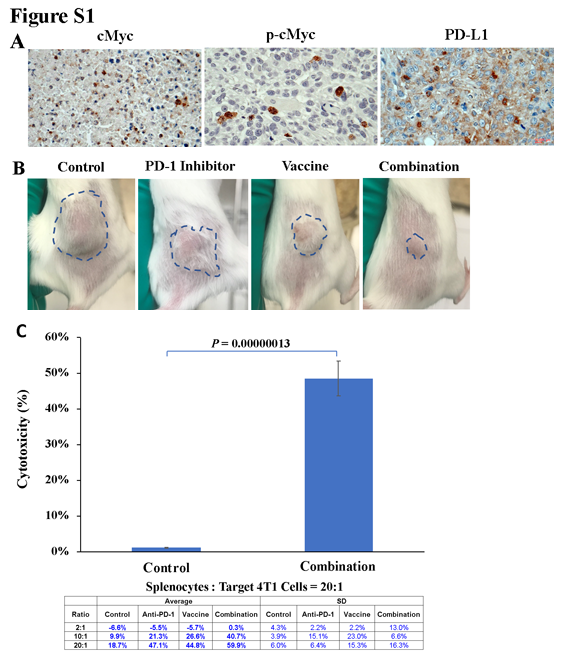

Supplement: Supplementary file 1 — Additional file 1: Figure S1. Effect of ASPH upregulation on PD-L1 expression. (A) PD-L1 upregulation was associated with ASPH-MYC signaling in BNL HCC derived tumors. (B) HCC tumor size at 49 days after implantation of BNL cells in different groups. ASPH-MYC signaling was observed in tumors derived from different groups. (C) In vitro cytotoxicity of splenocytes derived from mice of HCC model against 4T1 breast cancer target cells. [file 13046_2022_2307_MOESM1_ESM.tif]

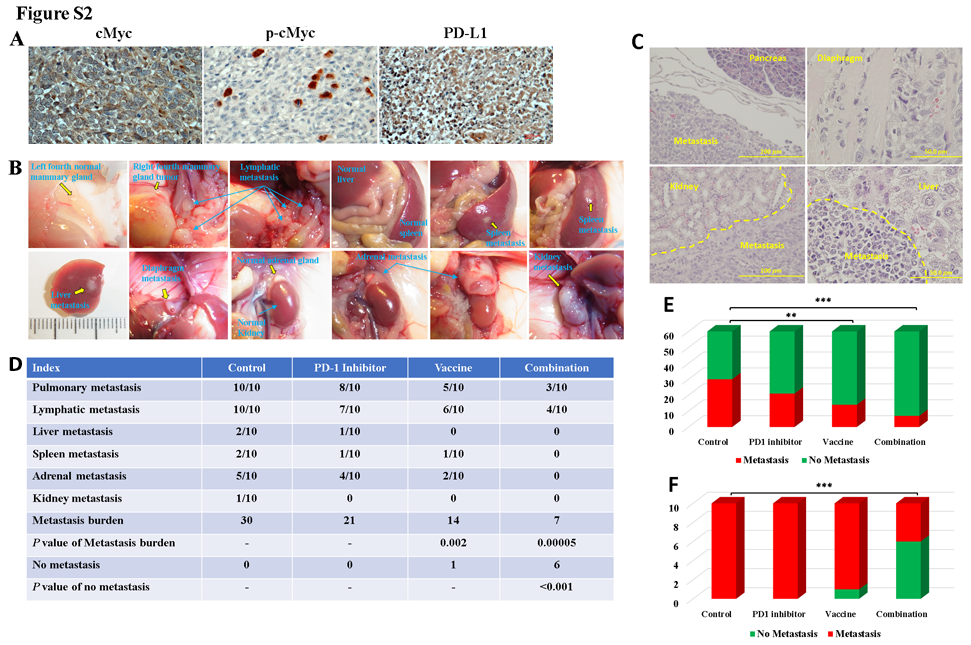

Supplement: Supplementary file 2 — Additional file 2: Figure S2. The TNBC exhibits an aggressive metastatic phenotype. (A) PD-L1 expression was associated with activation of ASPH-MYC signaling in TNBC cells. (B) Evidence of multi-organ metastases (lymph nodes, liver, spleen, pancreas, diaphragm, adrenal gland and kidney) in mice bearing TNBC tumors. (C) Representative histologic features of metastatic tumors. (D) Total number, (E) Frequency and (F) Distribution (number of mice with vs. without metastasis) of metastatic lesions or animals who developed metastases from different groups in TNBC model. *, p < 0.05; **, p < 0.01; ***, p < 0.005; ****, p < 0.001. [file 13046_2022_2307_MOESM2_ESM.tif]

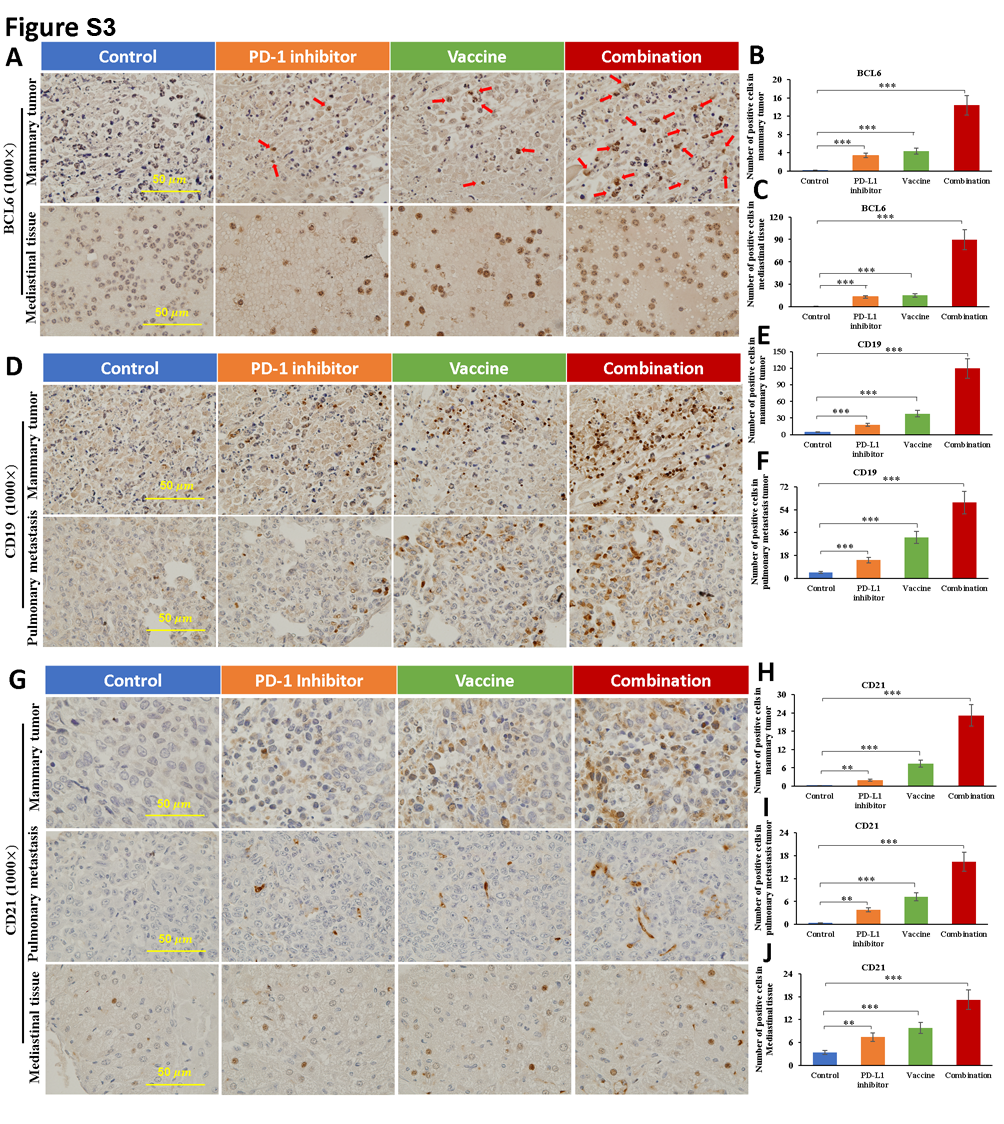

Supplement: Supplementary file 3 — Additional file 3: Figure S3. Intra-tumor TLS in 4T1 TNBC breast cancer tumors. (A-C) Number of CD4+/BCL6+ T follicular helper cell (TFH) population located in primary tumors and mediastinal metastases. (D-F) Number of CD19+ B population in primary tumors and pulmonary metastases. (G-J) Number of CD21+ follicular dendritic cell (FDC) population in primary mammary tumors as well as mediastinal and pulmonary metastases. *, p < 0.05; **, p < 0.01; ***, p < 0.005; ****, p < 0.001. [file 13046_2022_2307_MOESM3_ESM.tif]

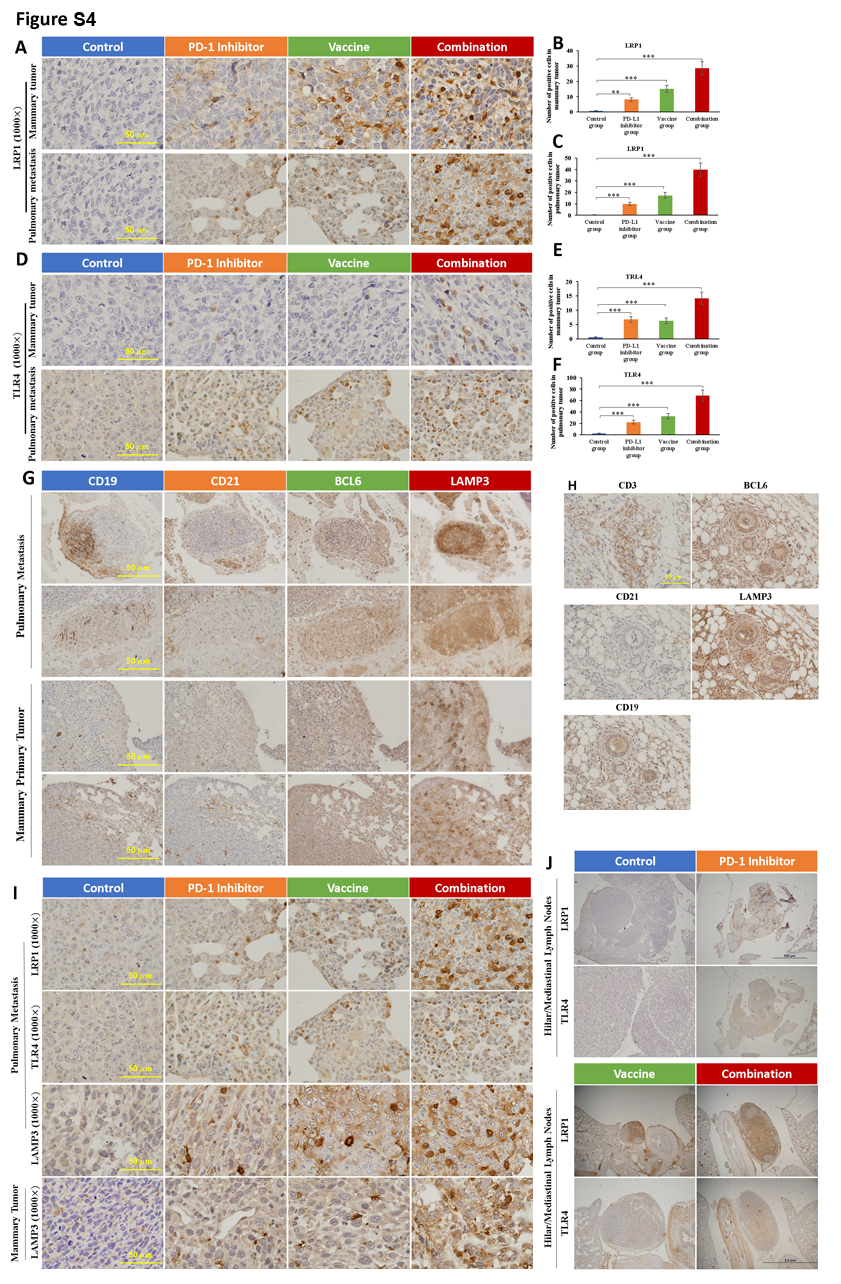

Supplement: Supplementary file 4 — Additional file 4: Figure S4. Activation and maturation of DCs are attributed to TLSs. (A-C) Number of LRP1+ DCs population in primary tumors and mediastinal metastases. (D-F) Number of TLR4+ DCs population in primary tumors and pulmonary metastases. (G) Intra-tumoral TLSs in primary mammary tumors and metastases induced by combination therapy. (H) Peri-tumoral TLSs (colocalized with CD3+ TILs) induced by combinatory therapy. (I) LAMP3+/LRP1+/TLR4+ DCs population in primary tumors and pulmonary metastases. (J) LRP1+/ TLR4+ DCs population in hilar/mediastinal lymph nodes metastases. *, p < 0.05; **, p < 0.01; ***, p < 0.005; ****, p < 0.001. [file 13046_2022_2307_MOESM4_ESM.tif]

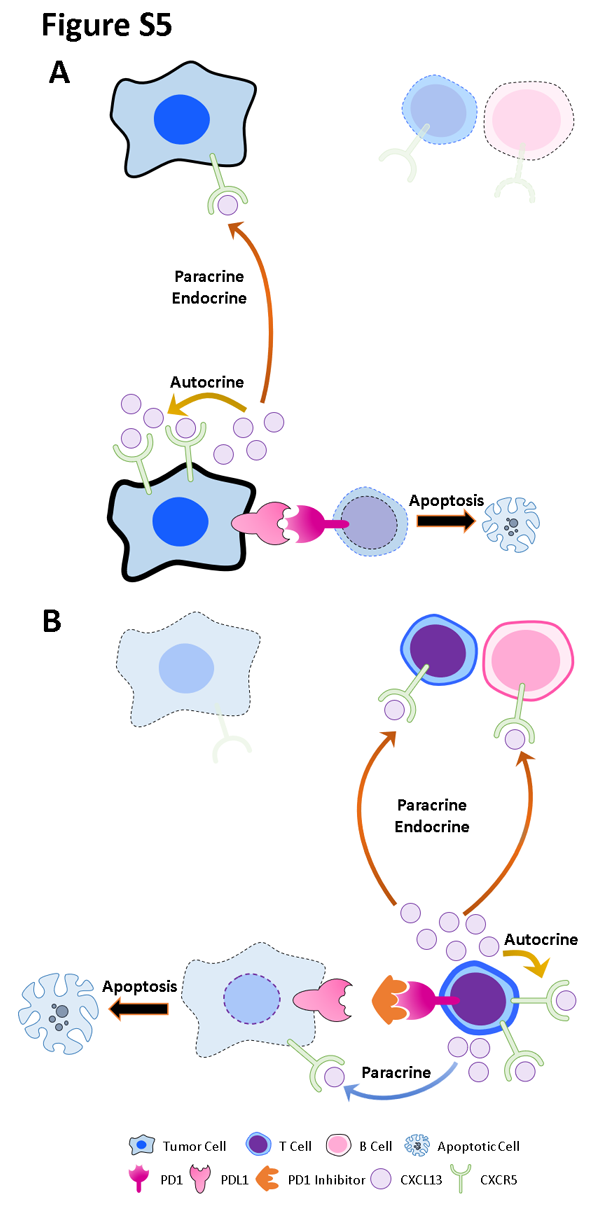

Supplement: Supplementary file 5 — Additional file 5: Figure S5. Hypothesized role of CXCL13/CXCR5 axis. (A) In the absence of PD-1 inhibitor, (autocrine, paracrine or endocrine) CXCL13 ligand secreted by cancer cells binding CXCR5 receptor to enhance aggressive phenotypes. Simultaneously, CXCL13 derived from cancer cells binding CXCR5 on T cells to aggravate PD1/PDL1 mediated T cell exhaustion, resulting in apoptosis of effector T lymphocytes. (B) In the presence of PD1 inhibitor, PD-L1 (on cancer cells)/PD-1 (on T cells) inhibitory signal is blocked. Effector T cells are activated and survive partially dependent on autocrine CXCL13/CXCR5. T cells secrete CXCL13 (paracrine or endocrine) to recruit CXCR5+ immune cells, thus initiating anti-tumor immune response. As attacked by CXCL13+ (paracrine) T cells, CXCR5+ cancer cells are subject to apoptosis. [file 13046_2022_2307_MOESM5_ESM.tif]
